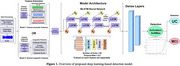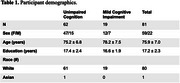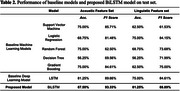# Detection of Cognitive Impairment Using Bidirectional Long Short‐Term Memory and Speech‐Derived Digital Biomarkers in Native English Speakers

**DOI:** 10.1002/alz70856_104560

**Published:** 2025-12-24

**Authors:** Pooyan Mobtahej, Sam T Gouron, Melina vom Saal, Dennis N Le, Tianchen Qian, Maria G Corona, Michelle McDonnell, David L Sultzer, Seyed Ahmad Sajjadi

**Affiliations:** ^1^ University of California, Irvine, Irvine, CA, USA; ^2^ Institute for Memory Impairments and Neurological Disorders, University of California, Irvine, Irvine, CA, USA

## Abstract

**Background:**

There is a crucial need for accessible and scalable methods of diagnosis for cognitive impairment and Alzheimer's disease. Our proposed deep learning‐based approach uses participants’ speech samples to compare acoustic and linguistic features to recognize early‐stage cognitive decline.

**Method:**

Participants of the Alzheimer's Disease Research Center (ADRC) at the University of California, Irvine, provided speech samples, which were processed and denoised to isolate participants’ speech. Of 162 audio samples (81 Picture Descriptions and 81 Spontaneous Speech), 80% were used for model training, 10% for validation, and 10% for testing; participants within each dataset were unique. We employed a Bidirectional Long Short‐term Memory (BiLSTM) model to capture patterns from both past and future data sequences. The model was applied independently for each feature set, one trained on acoustic features of speech and the other on linguistic features (Figure 1). The acoustic features consisted of Mel‐Frequency Cepstral Coefficients (MFCCs), Spectral Centroid, and Spectral Contrast, capturing the energy and quality of the speech audio; the linguistic features, extracted from transcriptions of participant speech samples, included vocabulary richness, average word length, sentiment score, and grammatical errors. Before input into their respective models, acoustic and linguistic features were transformed into 1D representations with 2‐fold cross‐validation to avoid overfitting. We ran the model to identify cognitive diagnosis as the outcome. Accuracy indicates the correctness of classification between mild cognitive impairment (MCI) and unimpaired cognition (UC) participants, while F1 score indicates overall model performance.

**Result:**

The study included 81 native English‐speaking participants (Table 1). 19 participants had MCI, and 62 participants had UC. Our proposed BiLSTM model achieved 87.50% accuracy with a 93.33% F1 score using acoustic features, and 81.25% accuracy with an 88.89% F1 score using linguistic features (Table 2). Our BiLSTM model outperformed machine learning and LSTM models in linguistic and acoustic features.

**Conclusion:**

Our results show that our proposed BiLSTM architecture outperforms machine learning and deep learning models, and that acoustic speech features are better predictors of cognitive impairment than linguistic features. The proposed model has the potential to improve the clinical diagnosis of cognitive impairment in an easy and scalable fashion.